# A Comparison of Biomechanical Properties of Implant-Retained Overdenture Based on Precision Attachment Type

**DOI:** 10.3390/ma14102598

**Published:** 2021-05-17

**Authors:** Małgorzata Idzior-Haufa, Agnieszka A. Pilarska, Wiesław Hędzelek, Piotr Boniecki, Krzysztof Pilarski, Barbara Dorocka-Bobkowska

**Affiliations:** 1 Department of Gerodontology and Oral Pathology, Poznan University of Medical Sciences, Bukowska Street 70, 60-812 Poznan, Poland; midziorhaufa@ump.edu.pl (M.I.-H.); bdorocka@ump.edu.pl (B.D.-B.); 2 Department of Plant-Derived Food Technology, Poznań University of Life Sciences, ul. Wojska Polskiego 31, 60-624 Poznan, Poland; 3 Department of Prosthodontics, Poznan University of Medical Sciences, Bukowska Street 70, 60-812 Poznań, Poland; whedzel@ump.edu.pl; 4 Department of Biosystems Engineering, Poznań University of Life Sciences, ul. Wojska Polskiego 50, 60-627 Poznan, Poland; bonie@up.poznan.pl (P.B.); pilarski@up.poznan.pl (K.P.)

**Keywords:** overdenture, bar, implant, mandible, finite element analysis

## Abstract

This paper aims to compare, in vitro, the biomechanical properties of an overdenture retained by two bar-retained implants and an overdenture retained by two bar-retained implants with ball attachments. An edentulous mandible model was prepared for the study based on the FRASACO mold with two implants. In the first system, the “rider” type (PRECI-HORIX, CEKA) retention structure and the complete mandibular denture with the matrix were made. In the second system, the “rider” type retention suprastructure was also used. In the distal part, (CEKA) clips were placed symmetrically, and a complete mandibular denture, together with the matrix on the bar, and the clip patrices were made. A numerical model was developed for each system where all elements were positioned and related to geometric relations, as in reality. The FEA analysis (finite element analysis) was carried out for seven types of loads: with vertical forces of 20, 50, and 100 N and oblique forces of 20 and 50 N acting on individual teeth of the denture, namely central incisor, canine, and first molar. Displacements, stresses, and deformations within the systems were investigated. Maximum denture displacement in the first system was 0.7 mm. Maximum bar stress amounted to 27.528 MPa, and implant stress to 23.16 MPa. Maximum denture displacement in the second system was 0.6 mm. Maximum bar stress amounted to 578.6 MPa, that of clips was 136.99 MPa, and that of implants was 51.418 MPa. Clips cause smaller displacement of the overdenture when it is loaded but generate higher stress within the precision elements and implants compared to a denture retained only by a bar. Regardless of the shape of the precision element, small deformations occur that mainly affect the mucosa and the matrix.

## 1. Introduction

Overdentures are one of the recognized methods of prosthetic treatment of the mandible. Relatively reasonable costs and uncomplicated clinical management with a significant improvement in retention and stabilization make this type of restoration an attractive treatment option for both patients and dentists [1,2,3]. Precision elements, such as bars, ball or locator clips, telescopic crowns, and magnets [4,5,6,7], limit denture mobility both vertically and horizontally. Precision attachments used with implant-retained overdenture counteract free rotation, so a greater occlusal force may be exerted because of the perceived better denture stability.

From the biomechanical point of view, overdentures are the optimal solution because they allow for a more uniform physiological distribution of chewing forces and their impact on the mucosa and alveolar process. Shortened teeth allow for a greater axial load. A variety of possible structural solutions of overdentures, concerning both the number and location of dental implants, as well as the types of clips, make rational justification for individual component application difficult. Despite the abundant amount of works in the literature, supraconstruction designs are often based on clinical tradition without addressing available theoretical background [8]. Consequently, along with other manufacturing errors or lack of follow-up care, unexpected and uncontrolled rotation of the denture, rapid wear, damage to its components, or implant overloading may happen [9].

The number of implants and placement sites depend on many factors, including anatomical conditions and the economic situation of the patient. Currently, many clinicians are inclined to use two implants. Opinions are more divided about the choice of a precision system. Two basic solutions include bar retention and single ball or locator attachments [10]. The choice of the precise element is an individual matter and depends on the conditions of the prosthetic area. When deciding on a bar retainer, we need to choose its proper shape and possible additional elements.

The modification of the bar is its extension, symmetrically, distally, beyond the implants. This results in better stability and reliable anchoring of the prosthesis to the bar. According to Mericske-Stern [11], the length of the cantilever bar may be up to approximately 1 cm and should not exceed the area of the first premolar. In turn, Elsyad [12] believes that the optimal length of the tabs is 7 mm. The smallest stresses are then generated without significant differences around the implants. Currently, instead of a cantilever bar, ball attachments are often used, placed on both sides, distally to the implants.

For a rational selection of the bar, suitable for a clinical situation, knowledge of its mechanical properties is indispensable to achieve long-term success in treatment [13]. In order to analyze the range of motion occurring within a denture and its retention element, it is required to find out the mechanical and fatigue characteristics of each component.

The aim of the study is to compare the in vitro biomechanical properties of an overdenture retained by two bar-retained implants with an overdenture retained by two bar-retained implants with ball clips.

## 2. Materials and Methods

A model of a toothless mandible based on the FRASACO mold with two OSTEOPLANT implants 14 mm long, and 4 mm in diameter was prepared for the study. The implants were located in the anterior region. Parameters of implant elements are shown in Table 1.

**Figure 1:**
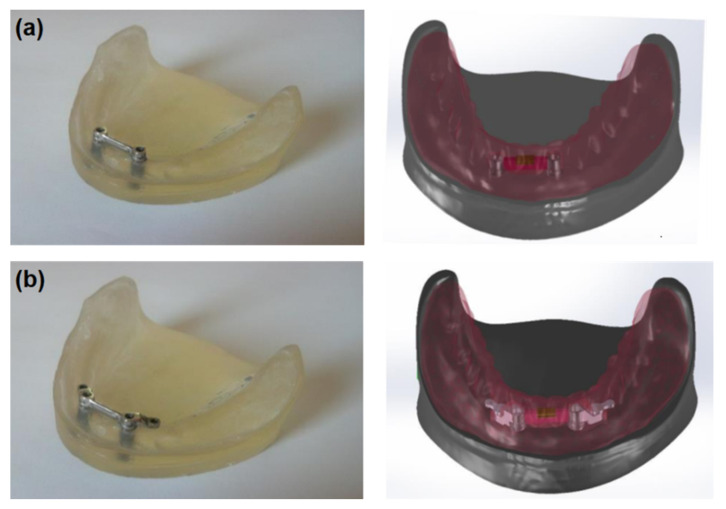
A real model and a computer model (**a**) of the first studied system and (**b**) of the second studied system.

**Figure 2:**
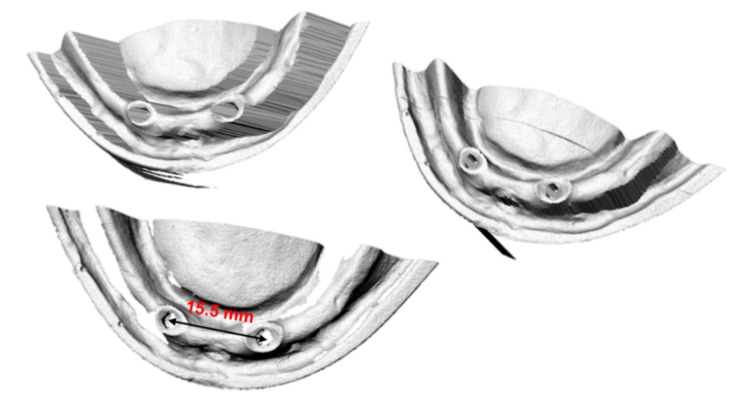
Visualization of measurement data obtained for the mandibular mucosa model in an LPX-600 scanner (in the real scale).

The first system included a “rider” bar type retention (PRECI-HORIX, CEKA) and the lower complete denture with a matrix (Figure 1a). In this clinical situation, a denture is primarily retained directly on the mucosa, while the bar provides adequate retention. In the second system (see Figure 1b), the “rider” bar type retention suprastructure was also used, and the ball clips (CEKA) were symmetrically placed in the distal part. A lower complete denture with the matrix on the bar and ball clip patrices was made, too (Figure 2). In this system, components are the same as in the first one; however, the method of fixing the denture is different. It is retained directly on the mucosa, and the bar’s function is to stabilize it, while the clips used are to retain the whole system.

The geometry of the mandible and dentures was scanned with the Roland LPX 600 3D Laser Scanner (Roland DGA Corporation, Tulsa, OK, USA) and imported into the computing environment. In order to increase accuracy, three independent measurements were made from three different directions (see Figure 2). Such a procedure eliminates places where the measuring head cannot register geometry during measurement from just one direction.

**Figure 3:**
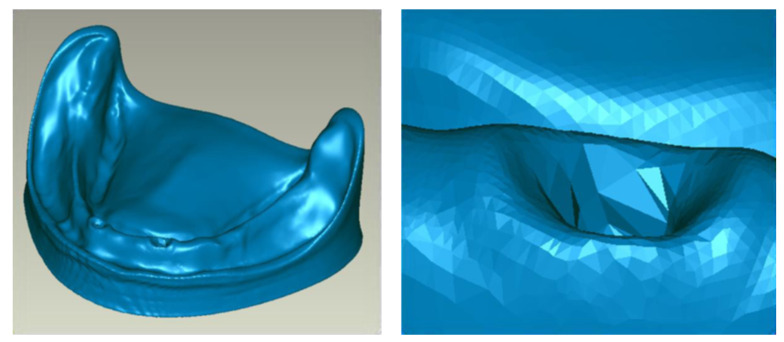
Mandibular mucosa geometry defined with a surface triangle mesh.

The data obtained by using a 3D (three-dimensional) scanner concerning, for instance, surface mesh, saved in an Standard Tessellation Language (STL) format, were transferred to the Geomagic Studio 12.0 software (Research Triangle Park, NC, USA) in which further processing was carried out. It consisted of matching and placing individual sets of measurement data on each other (3D scans) from three different directions so that one model with complete mandibular geometry (Figure 3) could be obtained. During this process, all data were filtered, the discontinuities of geometry were eliminated, and all points outside the mandible were removed (e.g., accidental elements of the background, the measuring table, etc.). Any surface distortions that arise during scanning and data placement were also removed during data processing.

**Figure 4:**
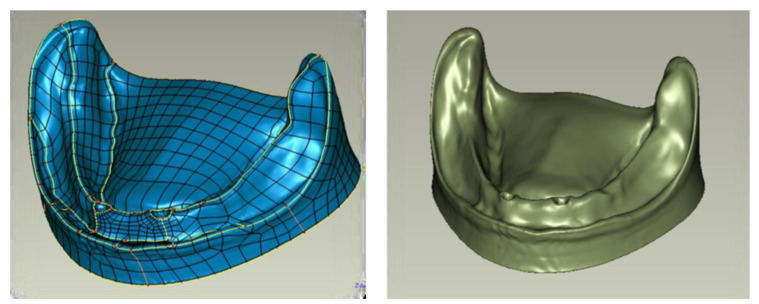
Division of mandibular mucosa into areas and final model defined with NURBS surfaces.

The obtained surface triangle mesh was subjected to further processing. Finite Elements Analysis (FEA) programs used for analysis require defining geometry as volumetric objects. For that, the next step involved changing the description of the mandibular mucosa geometry from surface triangle mesh to an object defined with Non-Uniform Rational Basis Spline (NURBS) type surfaces (see Figure 4).

To generate NURBS surface, contours along with division lines of particular areas of the mandible were determined. These measures allowed the surface formation of the model to be controlled well.

Next, a surface mesh of the object was formulated, taking into account division lines. Depending on the complexity of local geometry, it is possible to control mesh density. The obtained surface of the mandibular mucosa defined with NURBS was exported by using a universal computer-aided design (CAD) data exchange file.

**Figure 5:**
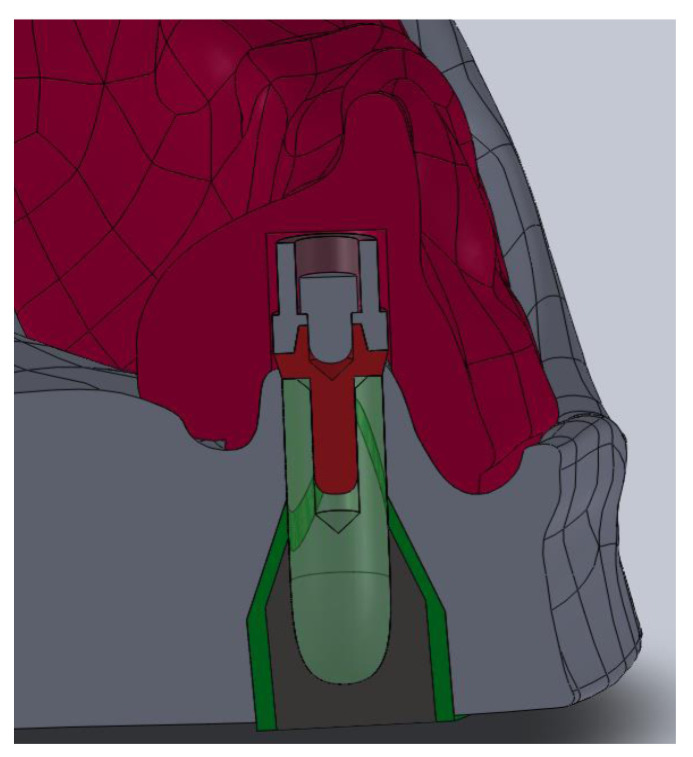
A cross-section showing the connection of system elements with bone.

The other system components (including implants, conical abutments, bar, etc.) were modeled directly in the SolidWorks software—a CAD program, version 2013 (Dassault Systems Co., Waltham, MA, USA)—because, in the computed tomography (CT) scan and scanner image, metal elements produce a light artifact in the form of a flare, which makes the boundary of the object unclear. Geometric data of individual components were obtained from the manufacturer’s documentation and from our measurements. Spacing was positioned from CT image, scanner, and control measurements. To simplify numerical calculations, the mapping of threaded connections (outline of the helical thread line) was abandoned due to their negligible significance for the study. It would considerably prolong the calculation time, too. Ankylotic connection of the implants with the bone was assumed; that is, the thread connection was considered ideal and, hence, deprived of any mobility. Compact and spongy bone were matched to the model of the mandible with respect to the mucous membrane. A denture was developed with a 3D scanner, and data processing was analogous to the one done in the mandible modeling. The denture was adapted to the geometry of the mandible. An ideal adhesion of the denture to the prosthetic bearing area was assumed. All elements were positioned and geometrically related, as in reality (Figure 5).

Parameters of the oral mucosa thickness and compact and spongy bone thickness were determined by following the literature data. For the mucous membrane, a thickness of 1.5 mm was assumed, Young’s modulus E = 5 MPa and a quite high Poisson’s ratio ν = 0.49, which to some extent imitated its incompressibility [14,15]. For the cortical bone, Young’s modulus E = 17 GPa was assumed; for spongy bone, E = 600 MPa with Poisson coefficient equal to 0.3 in both cases. Material features of the denture are described with E = 3500 MPa and ν = 0.35 data [16,17].

Values of basic material properties of individual elements determined based on the literature are shown in the table (see Table 2) [14,15,18,19].

The prepared model was exported to the FEA calculation module. Finite element analysis enables evaluating the distribution of internal forces in a given object when exposed to an external load. In the loaded construction elements, there are two states: stress (loading force divided by a cross-section area, e.g., of an implant) and deformation, a change in an element geometry. When these two states are fused in one mathematical formula, a mathematical model is formulated, e.g., elasticity [20,21,22].

FEA was performed in the SolidWorks Simulation calculation module of the SolidWorks program. FEA mesh parameters for systems I and II are given in Table 3.

Forces of 20 and 50 N were applied at 0° and 45° angles, as well as 100 N at 0° angle [16,17,23,24]. These forces correspond to the average and maximum loads on the teeth in occlusion. It should be added that, in patients using removable dentures, lower forces are produced during chewing. The range of occlusive forces from 20 to 90 N allows us to obtain a satisfactory mechanical efficiency for the denture. The maximum acceptable values of occlusive forces can be up to 122 N [25]. The tests simulated chewing loads when the denture is loaded not only in the vertical but also in the horizontal plane. In the first case, the force was applied to the right central incisor at an angle of 0° (P1); in the second case, the same tooth was loaded with the forward force at 45° (P2). In the third and fourth cases, the force was directed to the right canine. In the third case, the force was applied at 0° (P3); in the fourth one, at 45°, perpendicular to the tooth (P4). In the fifth, sixth, and seventh cases, the force was directed to the right first molar. In the fifth, the force was applied at 0° (P5); in the sixth, at 45°, laterally toward the cheek (P6); and in the seventh, at 45°, forward (P7).

The displacements were in three directions: XYZ and net forces, stress reduction according to von Mises, and deformations were investigated. Displacement is a change in a given point position, i.e., a difference between initial and final position, e.g., extension, bending, and distortion angle. Its units are meters (m) or degrees (°) [20,21,22].

To speak about displacement, one has to determine an object (a system) state called an undeformed state. It is an arbitrary state where the position of all points of an object is known. It means that the following function is known (1):

(1) $$\vec{R_{0}}:\Omega \to P$$

where $\vec{R_{0}}$ is the position vector, Ω is a set of all object points, and P is a set of all space points in which an object is “suspended”. In practice, such a state occurs when no force is applied to an object. Considering other object states, it is possible to determine its points position $\vec{R_{1}}$. In light of the above, displacement is expressed as the following subtraction (2):

(2) $$\vec{u}=\vec{R_{1}}-\vec{R_{0}}$$

It means that displacement is a vector field that is assigning each object point its displacement vector.

To evaluate stress, a modified von Mises criterion was applied. According to it, the material will be damaged when reduced stress exceeds tensile strength [26]. Contour maps show the distribution of stresses obtained in computer simulations.

Deformation, or relative deformation, is the ratio of displacement to initial dimensions, for instance, due to tension:

(3) $$\varepsilon =\frac{\Delta l}{l_{0}}=\frac{l_{k}-l_{0}}{l_{0}}$$

where *l*_0_ is the initial length, and *l_k_* is the final length.

It is a dimensionless quantity (or %) [22,23,24,25,26].

The obtained results were analyzed and graphically presented in tables, graphs, and contour maps.

## 3. Results

**Figure 6:**
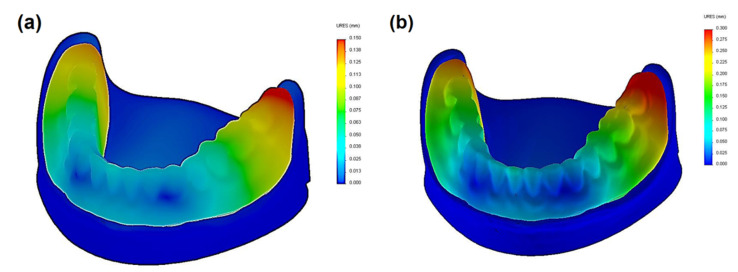
Distribution of displacements in an overdenture when an incisor is loaded with a vertical force of 50 N: (**a**) system I and (**b**) system II.

**Figure 7:**
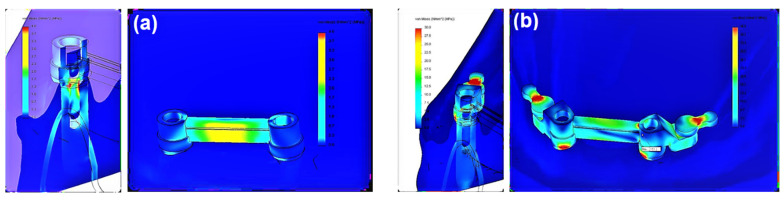
Distribution of stress within an implant and precision element when an incisor is loaded with a vertical force of 50 N: (**a**) system I and (**b**) system II.

**Figure 8:**
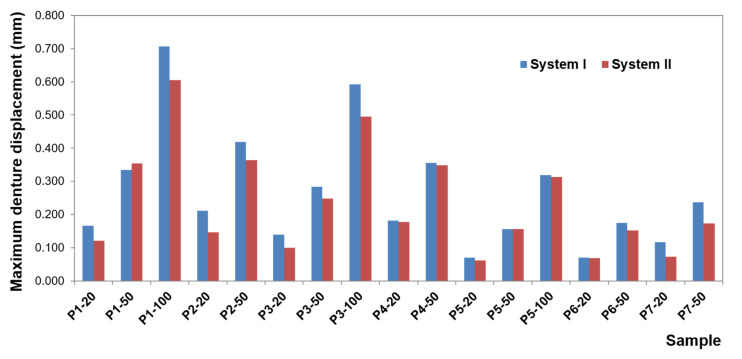
Maximum denture displacement in the first and second system.

**Figure 9:**
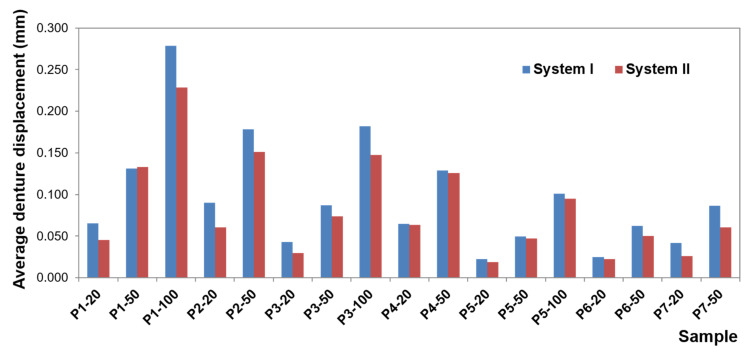
Average denture displacement in the first and second system.

**Figure 10:**
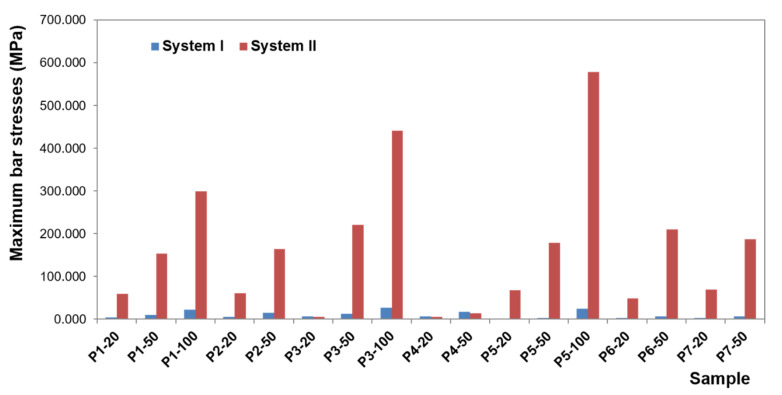
Maximum bar stresses in the first and second system.

**Figure 11:**
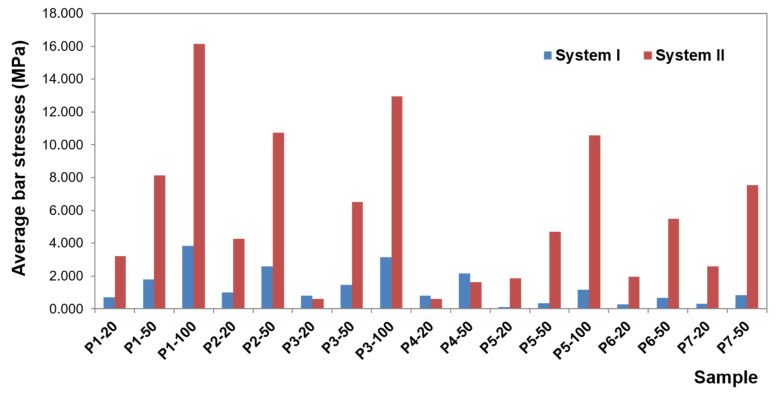
Average bar stresses in the first and second system.

**Figure 12:**
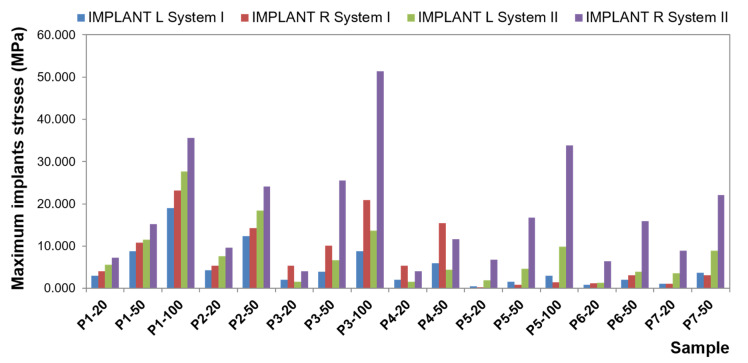
Maximum implant stresses in the first and second system (explanation: L—left; R—right).

To present the results, a force load of 50 N was selected. Contour maps show places where the greatest stress is concentrated within the bar retention and clips, as well as around the implants (Figure 6). The places where the denture undergoes the most significant displacements are also depicted (Figure 7). Crucial results obtained in the tests, i.e., displacement of the denture and the stresses of the bar and implants, are shown in the graphs (Figure 8, Figure 9, Figure 10, Figure 11 and Figure 12).

Greater dislocations of the denture were recorded in the first system, where the maximum value was 0.7 mm (Figure 8). In the second system, the maximum displacement of the denture was 0.6 mm (Figure 9). The largest displacements occur when an incisor is loaded. For forces of the same value, greater displacements occur for oblique forces. Regardless of the location of the load, the most significant dislocations occur in the retromolar pad region. With forces acting on an incisor, especially for oblique forces, displacements are found on both sides of the denture.

For forces acting on a canine and molar, the denture undergoes greater displacement on the balancing side. Depending on the shape of the precision element used, the value of displacement changes while the direction of rotation remains the same. Loading of the examined teeth results in rotational motion on the working side, pressing against the denture bearing area, and tilting from the YZ plane (~in the XY plane, ~around the Y-axis) on the unloaded side, which results in detaching the denture on this side. The use of CEKA clips results in extension of the retention element, which results in a more stable denture, less significant displacement, especially when loading an incisor and canine. Adverse effects of oblique forces are also smaller, and there is no rotation. Mucosal load onto the bone remains smaller, too. The use of CEKA clips makes the bar itself subjected to greater displacement, especially with forces directed to an incisor, which may bring about a larger bone load around the implants. When exposing the denture to a single load, no significant differences were found in the bone, both cortical and spongy, for individual bars. However, tests should also include cyclic loads.

CEKA clips are part of a system that is exposed to displacements, especially when loading a canine and incisor. It should be noted that oblique forces are always less favorable for a clip on the working side. However, when loading canine for the clip on the balancing side, greater displacements occur for vertical forces. The obtained data show that implants and conical abutments make very stable system elements, regardless of the bar used.

The weakest element of this part of the system is the place where the bar is fixed, i.e., the screws, especially when loading a molar with both a vertical force of 100 N and oblique forces. For these forces, greater displacements within the screws occur on the balancing side than on the working one. When a denture retained by a “rider” bar is loaded with a vertical force of 100 N, and the displacement of the screws exceeds the displacement of the denture. However, the use of CEKA clips significantly limits the screw displacement, leading to protection against loosening.

The greatest stress concentrate within the precision element and concern the bar. In the first system, bar stress ranges from 0.0020 to 27.552 MPa (Figure 10), obtained when a canine is loaded with a vertical force of 100 N. High values of maximum stresses when loading all teeth with a vertical force of 100 N are, however, focal, while the average values are definitely smaller (3.8260 MPa for an incisor, 3.1607 MPa for a canine, and 1.1483 MPa for a molar). Regardless of the force location, higher stresses occur for oblique forces. The largest bar stresses appear in the medial area, especially for the incisor and canine. For the molar, stresses are distributed over a larger area. Moreover, during loading of the molar, there are clearly lower stresses in the bar than in an incisor or canine, and the force of 50 N does not exceed 1 MPa. In the second system, bar stresses range from 0.080 to 578.6 MPa (Figure 11). The highest stresses occur when loading a molar tooth with a vertical force of 100 N. For an incisor and molar, higher stresses occur for oblique forces (for a force of 50 N, average bar stresses for an incisor are 8.1340 MPa for vertical force and 10.7370 MPa for oblique force; for a molar, they are 4.7082 MPa for vertical force and 5.4998 and 7.5317 MPa for oblique forces). Relatively small stresses arise in the bar when loading a canine with oblique forces (for the force of 50 N, the maximum value is 13.4736 MPa, and average 1.6251 MPa). In the second system, the precision element was extended by mounting the CEKA clips. The maximum stresses produced in clips are smaller than the stresses of the bar. Stress values range from 0.0538 to 136.9900 MPa for the right clip and from 0.0784 MPa to 127.6700 MPa for the left. The highest stresses occur in the right clip with a vertical force of 100 N applied on a canine and in the left clip when applying this force on an incisor. Significant maximum values of clip stresses result from their shape and are located within the narrowing. CEKA clips cause significantly higher stresses in the screws, conical abutments, and implants than when a bar is used alone.

Comparative analysis of the first and second system (Figure 12) shows that the additional use of CEKA clips causes bar extension, and therefore the denture is less susceptible to displacement. Still, more stresses are generated within the bar. Both the value of the maximum and average stresses is several times greater. The distribution of stresses is changed, too. When a bar is used alone, the greatest stresses are limited, located in the medial area. CEKA clips result in a more even distribution of stresses in the bar, with a greater concentration of stresses in clips. Significant stresses in this area are also associated with the clip shape. The material narrowing site causes a notch effect. Interestingly, oblique forces applied to all teeth give rise to similar stresses in both clips. An exception is an oblique force that is directed forward to a molar and thus acting in the final phase of the chewing cycle, where higher stresses are on the balancing side. For vertical forces, the greatest stresses appear on both sides when loading an incisor. Loading a canine generates higher stresses on the working side, whereas loading a molar causes relatively smaller stresses, which are asymmetrical and greater on the balancing side.

For subsequent components of the precision element and implants, the stresses are smaller and smaller because each element has absorbed part of the energy created by the load. Of course, ideally, stresses for this part of the system would equal 0. However, the material applied for testing is real. These elements work, and the denture works; hence, there are low values of stress. It is essential that CEKA clips cause a significant increase in stresses within conical abutments and screws. Proportionally higher stresses in the screws when using CEKA clips result from the screws being more stable in this system, as they undergo lesser displacement. Hence, a smaller risk of loosening. However, an increase in stress may cause mechanical damage to be more likely. Higher stresses in conical abutments and screws generate lower stresses in the implants, too.

**Figure 13:**
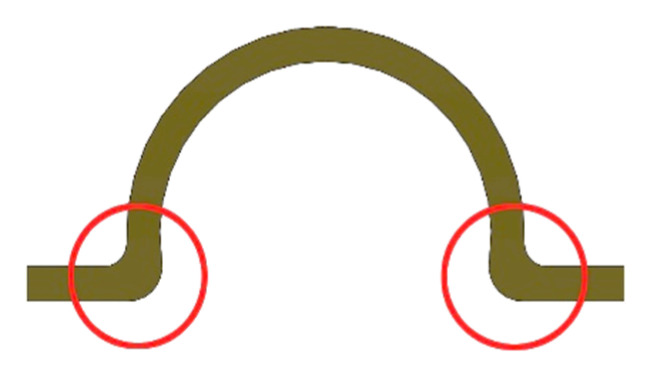
Stress concentration in matrix housing.

Moreover, the studies showed that significant stresses were observed for the matrix housing in the first and the second system. However, maximum values occurred only in the site marked in Figure 13. In other places, stress values are smaller. The place where maximum stress occurs is the result of element geometry, causing stress buildup in this area. This place can be exposed to mechanical damage, but due to embedding in acrylic, it will be difficult to locate, but it does not necessarily affect the denture function.

High values of stress were found in the denture. Maximum values, however, occur only in the place where the force is applied. This situation is not clinical in nature, as under physiological conditions, the load is applied to the denture plane and is not point-wise. The average values of stress are incomparably smaller. Generally, there are no significant stresses in the denture as it undergoes dislocations rather than stresses when loaded.

**Figure 14:**
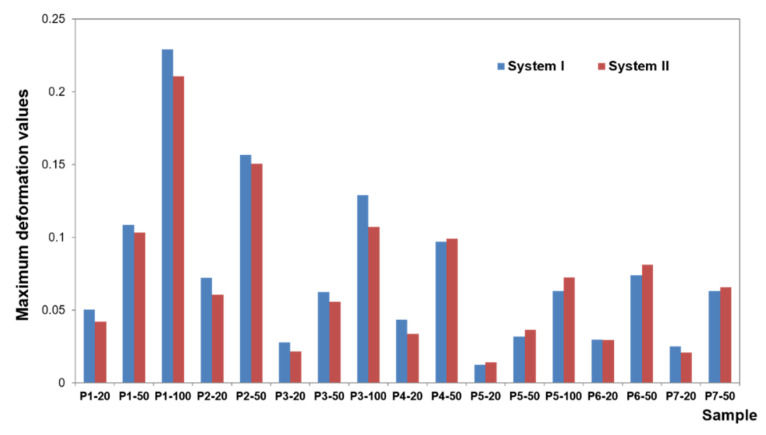
Maximum mucosa deformations for systems I and II.

**Figure 15:**
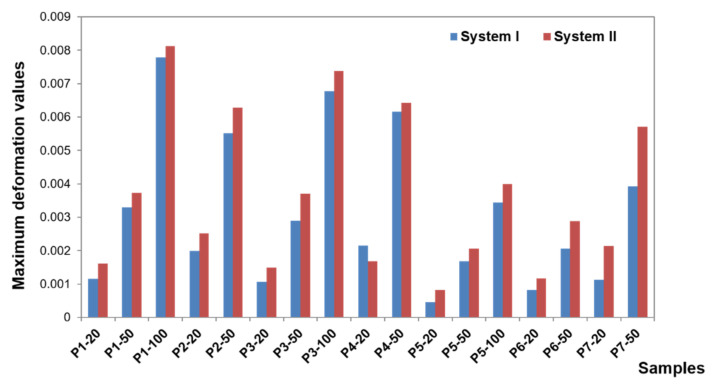
Maximum matrix deformations for systems I and II.

The deformation results concerning each case show that the subsequent components undergo only minor deformations, which are not relevant for understanding the biomechanics of the overdenture. During the deformation analysis, only the mucosa and the matrix together with the housing deserve attention. Irrespective of the shape of the precision element, the highest mucosal deformations occur when an incisor is loaded (Figure 14). In system I, the maximum deformation values are 0.22900 for a vertical force of 100 N and 0.15670 for an oblique force of 50 N. The use of CEKA clips additionally does not significantly change the deformation values, which are maximum at 0.21060 for a vertical force of 100 N and 0.15060 for an oblique force of 50 N. The results concerning the first and second system show that the mounting of CEKA clips does not significantly affect the deformation values of the matrix and its housing, causing only a slight increase, especially when loading the molar tooth (Figure 15). The highest deformation values were recorded for the forces directed at the incisal tooth, and when only the bar is used, they are 0.0144 (matrix housing) and 0.0077 (matrix) for a vertical force of 100 N and 0.0057 and 0.0055, respectively, for an oblique force of 50 N. However, when the bar with CEKA clips is used, the maximum deformations are 0.0109 and 0.0081 for a vertical force of 100 N and 0.0066 and 0.0062, respectively, for an oblique force of 50 N.

Denture deformations occur mainly at the force application site. Similar deformation values were found for both systems. In system I, the largest deformations occur for vertical forces when the molar is loaded, namely 0.09744 (for 100 N force), and for oblique forces when the incisor is loaded, namely 0.0490 (for 50 N force). In system II, the largest deformations occur for vertical forces when loading the canine, namely 0.08876 (for 100 N force), and for oblique forces when loading the incisor, namely 0.1506 (for 50 N force).

Regardless of the shape of the precision element, the denture undergoes little deformation. Acrylic is a rigid material so that the denture is displaced under load but not deformed. As expected, the element that undergoes significant deformation is, due to its highest elasticity, the mucosa. The deformation values of the mucosa are related to the displacement of the denture. Another element where increased deformation was observed is the matrix, which is the second most elastic part. For both systems, however, the deformation values are already much smaller. Larger values of deformation of the matrix in relation to the other components of the model, related to its elasticity, are the cause of wear of this element under the influence of the use of the denture.

## 4. Discussion

A significant problem when planning an overdenture is the choice of an appropriate precision system. Many researchers consider dentures retained by constructions splinting to be an advantageous solution [27,28,29]. Undoubtedly, the advantages of bar constructions include proper denture stabilization and the ability to regulate retention.

Loads during denture dislocations in the lateral region are reduced [30]. The incidence of complications is also lower compared to ball attachments. Magnets, which are less frequently used, can corrode and allow for horizontal displacement of the denture [31]. They also have the lowest retention strength. It should be remembered, however, that bars require more space occlusally, that is, between the jaws (13–14 mm), as compared to ball attachments (10–11 mm) [32]. No adequate space may result in the incorrect shape of the denture plate or the position of artificial teeth. This, in turn, may bring about faulty facial aesthetics or impaired sound articulation [33,34,35,36,37].

The studied bar has a retention shape. Currently, connections of round or straight cross-sections are less and less often used. These bars are only indicated for short implants or problems with osseointegration, as they generate fewer stresses around the implants [38]. To improve retention, the “rider” bar is better. The tests have confirmed that the more retentive the shape of the precision element, the smaller the denture dislocations. The length of the connection is of great importance. The greater the length of the retention arm, the more rigid the denture. Higher rigidity of the prosthetic restoration causes smaller displacement of the restoration, so mucosal support of the denture is smaller as well. However, the stresses within the precision element and implants are greater. The site of implant insertion may determine the length of the retention arm. The wider the distance between implants, the longer the connection. However, it should be remembered that when the line connecting the implants exceeds the denture plate, only individual clips are possible [37]. To extend the retention arm, it is possible to use symmetrical projections or clips distally. The results of the studies show that the use of additional clips reduces denture displacement. However, the bar with clips and implants is more loaded; the more retentive the shape of the precision element, the greater the stresses [38,39].

In the case of short implants and substantial bone atrophy, it seems more advisable to make a straight bar to increase the mucosal force transfer and relieve the implants. Studies showing that a longitudinal bar extended distally (with a twice-broken axis) causes a significant exceeding of the stress tolerated by the bone tissue can be found in the literature. Still, this study has not confirmed such a phenomenon. An increase of stress in this area was observed only, which in extreme cases cannot rule out bone resorption around the implant. A relatively smaller stress increase in the implants is related to the fact that conical abutments and screws absorb a greater part of the energy released from loading forces. This is where a marked increase in stress was noted. It seems, therefore, that the risk of loosening or even damage to these elements is greater than the disintegration of the implant or peri-implantitis [40,41]. The conducted research confirmed the reports of other authors that the greatest load occurs in the distal parts of the bar retention [42,43]. Stress concentration, however, within the clips results from their shape.

The implementation of additional clips is indicated for long implants in the absence of clinical problems with osseointegration. Then, the use of direct support on implants that are ankylotically connected to the bone relieves the structure of the denture bearing area, especially in case of a mucous membrane poor susceptibility. This contributes to protecting the underlying tissues, which are not physiologically adapted to sustain direct occlusal forces, which may lead to bony atrophy in a short time [44,45].

The studies presented in this paper show the interdisciplinary character of today’s prosthetics. FEA is applied by a growing number of researchers [24,38,43,46]. Advances in virtual engineering enable making computer simulations in concrete and stable conditions, which helps avoid mistakes in experimental studies. Of course, it would be prudent to perform an experimental validation to verify the result. Confirmation of the FEA ideally would be performed in human subjects, but that is very difficult to perform stress tests in vivo. For this test, a phantom can be prepared, but it should be noted that it is a very complicated biological model with heterogeneous materials. Nevertheless, in FEA studies on dental implants, validation techniques are still rare. In his study, Chang performed a literature review of finite element analysis of dental implants. In PubMed, he found 522 articles about FEA, but only 47 papers including validation [47].

In the near future, the authors of this study plan to compare the results obtained in the presented article with clinical trials.

## 5. Conclusions

1. The shape of a precision element affects the biomechanical properties of an overdenture.
2. Slight stresses are observed within the studied bar-retained precision attachment connected to conical abutments and implants.
3. Ball clips cause less displacement of an overdenture when loaded. However, they generate higher stresses within the precision element and implants, compared with the overdenture retained on the bar-retained implants only.
4. Regardless of the shape of the precision element, small deformations occur, which mainly affect the mucosa and the matrix.
5. For the analyzed situation, the best solution seems to be using the bar with CEKA clips.

## Figures and Tables

**Table 1:** Implant element parameters.

	Platform	Diameter (mm)	Height (mm)	Material
BASE implant	ND	4.0	14	Ti4
Conical abutment	M	4.5	1.0	Ti 23
Conical abutment screw	ND	ND	ND	ND
Technical cap	ND	ND	ND	POM

ND—not determined; POM—poly(methylene oxide).

**Table 2:** Material properties.

Part	Young’s Modulus (GPa)	Poisson’s Ratio (-)	Density (kg (m^3^)^−1^)	Plasticity Boundary (N (mm^2^)^−1^)
Denture	3.5	0.35	1190	ca 17.5
Matrix housing	200	0.26	517.02	206.81
Matrix	1.4–7	0.39	1330–1610	45–65
Screw	110.3	0.31	4480	744.63
Bar	2	0.3	8900	827
Conical abutment	113.8	0.342	8900	827
Implant	105.2	0.37	4510	500
Mucosus membrane	0.005	0.49	1000	9.5
Compact bone	17	0.3	1900	48
Spongy bone	0.6	0.3	380	48
Clip	113.8	0.342	8900	827

**Table 3:** FEA mesh parameters for systems I and II.

Parameter Type	System I	System II
Studied element	Bar 1	Bar 2
Mesh type	Tri-dimensional mesh	Tri-dimensional mesh
Mesh generator used	Standard mesh	Curvature-based mesh
Jacobian	4 points	4 points
Mesh control	Defined	Defined
Element size	1.5 mm	ND
Maximal element size	ND	7.86174 mm
Minimal element size	ND	1.25788 mm
Tolerance	0.02 mm	ND
Mesh quality	High	High
Total number of knots	659,329	784,006
Total number of elements	431,348	518,639
Maximal shape coefficient	263.08	509.35
Shape coefficient < 3	95.9	97.6
Shape coefficient > 10	0.23	0.12

ND: not determined.

## Data Availability

We do not provide additional data.

## References

[1] Keshk A.M., Alqutaibi A.Y., Algabri R.S., Swedan M.S., Kaddah A. (2017). Prosthodontic maintenance and peri-implant tissue conditions for telescopic attachment-retained mandibular implant overdenture: Systematic review and meta-analysis of randomized clinical trials. Eur. J. Dent..

[2] Ekelund J.A., Lindquist L.W., Carlsson G.E., Jemt T. (2003). Implant treatment in the edentulous mandible: A prospectiv study on Branemark system implants over more than 20 years. Int. J. Prosthodont..

[3] Schmidt M.B., Rauch A., Schwarzer M., Lethaus B., Hahnel S. (2020). Combination of digital and conventional workflows in the CAD/CAM-Fabrication of an implant-supported overdenture. Materials.

[4] El-Anwar M.I., El-Taftazany E.A., Hamed H.A., ElHay M.A.A. (2017). Influence of Number of Implants and Attachment Type on Stress Distribution in Mandibular Implant-Retained Overdentures: Finite Element Analysis. Maced. J. Med. Sci..

[5] Michelinakis G., Barclay C.W., Smith P.W. (2006). The influence of interimplant distance and attachment type on the retention characteristics of mandibular overdentures on 2 implants: Initial retention values. Int. J. Prosthodont..

[6] Uçankale M., Akoğlu B., Ozkan Y., Ozkan Y.K. (2012). The effect of different attachment systems with implant-retained overdentures on maximum bite force and EMG. Gerodontology.

[7] Liu W., Zhang X., Qing H., Wang J. (2019). Effect of LOCATOR attachments with different retentive forces on the stability of 2-implant-retained mandibular overdenture. J. Prosthet. Dent..

[8] Sadowsky S.J. (2019). Occlusal overload with dental implants: A review. Int. J. Implant. Dent..

[9] Dorocka-Bobkowska B., Medyński D., Pryliński M. (2017). Recent advances in tissue conditioners for prosthetic treatment: A review. Adv. Clin. Exp. Med..

[10] Cicciù M., Tallarico M. (2021). Dental implant materials: Current state and future perspectives. Materials.

[11] Mericske-Stern R., Taylor T., Belser U. (2000). Management of the edentulous patient. Clin. Oral Implants Res..

[12] Elsyad M.A., Al-Mahdy Y.F., Salloum M.G., Elsaih E.A. (2013). The effect of cantilevered bar length on strain around two implants supporting a mandibular overdenture. Int. J. Oral Maxillofac. Implants.

[13] Mazaro J.V., Filho H., Vedovatto E., Pellizzer E., Rezende M.C., Zavanelli A. (2011). Evaluation of stress patterns produced by implant-retained overdentures and implant-retained fixed partial denture. J. Craniofac. Surg..

[14] Chun H.J., Park D.N., Han C.H., Heo S.J., Heo M.S., Koak J.Y. (2005). Stress distributions in maxillary bone surrounding overdenture implants with different overdenture attachments. J. Oral. Rehabil..

[15] Chowdhary R., Lekha K., Patil N.P. (2008). Two-dimensional finite element analysis of stresses developed in the supporting tissues under complete dentures using teeth with different cusp angulations. Gerodontology.

[16] Żmudzki J., Chladek G., Kasperski J. (2015). Biomechanical factors related to occlusal load transfer in removable complete dentures. Biomech. Model Mechanobiol..

[17] Tanaka M., Ogimoto T., Koyano K., Ogawa T. (2004). Denture wearing and strong bite force reduce pressure pain threshold of edentulous oral mucosa. J. Oral Rehab..

[18] Prados-Privado M., Martínez-Martínez C., Gehrke S.A., Prados-Frutos J.C. (2020). Influence of bone definition and finite element parameters in bone and dental implants stress: A literature review. Biology.

[19] Żmudzki J., Chladek W. (2010). Identification of biomechanics related to single implant-retained tissue-supported dentures. Prosthodontics.

[20] Budynas N., Nisbett K. (2008). Shigley’s Mechanical Engineering Design.

[21] Dowling N.E. (2018). Mechanical Behavior of Materials: Engineering Methods for Deformation, Fracture, and Fatigue.

[22] Gere J.M. (2019). Mechanics of Materials, Enhanced.

[23] El-Anwar M.I., Yousief S.A., Soliman T.A., Saleh M.M., Omar W.S. (2015). A finite element study on stress distribution of two different attachment designs under implant supported overdenture. Saudi Dent. J..

[24] Assuncao W.G., Tabata L.F., Barao V.A.R., Rocha E.P. (2008). Comparison of stress distribution between complete denture and implant-retained overdenture-2D FEA. J. Oral Rehab..

[25] Lee J.H., Kim W.H., Shin R.H., Lee K.W. (2008). A comparison of the masticatory function between two different types of implant supported prostheses and complete denture for fully edentulous patients. J. Korean Acad. Prosthodont..

[26] Dejak B., Mlotkowski A. (2008). Three-dimensional finite element analysis of strength and adhesion of composite resin versus ceramic inlays in molars. J. Prosthet. Dent..

[27] Schierano G., Manzella C., Menicucci G., Parrotta A., Zanetti E.M., Audenino A.L. (2015). *In vitro* standardization of two different removal devices in cemented implant prosthesis. Clin. Oral Implants Res..

[28] ElSyad M.A., Alameldeen H.E., Elsaih E.A. (2019). Four-implant-supported fixed prosthesis and milled bar overdentures for rehabilitation of the edentulous mandible: A 1-year randomized controlled clinical and radiographic study. Int. J. Oral Maxillofac. Implants.

[29] Savabi O., Nejatidanesh F., Yordshahian F. (2013). Retention of implant-supported overdenture with bar/clip and stud attachment designs. J. Oral Implantol..

[30] Takeshita S., Kanazawa M., Minakuchi S. (2011). Stress analysis of mandibular two-implant overdenture with different attachment systems. Dent. Mater. J..

[31] Kokubo Y., Fukushima S. (2002). Magnetic attachments for esthetic management of an overdenture. J. Prosthet. Dent..

[32] Gajdus P., Rzątowski S., Idzior-Haufa M. (2013). Overdentures supported by Osteoplant implants in difficult types of prosthetic area. Prosthodontics.

[33] Osman R., Payne A., Ma S. (2012). Prosthodontic maintenance of maxillary implant overdentures: A systematic literature review. Int. J. Prosthodont..

[34] Mackie A., Lyons K., Thomson W., Payne A. (2011). Mandibular two-implant overdentures: Three-year prosthodontic maintenance using the locator attachment systems. Int. J. Prosthodont..

[35] Andreiotelli M., Att W., Strub J.R. (2010). Prosthodontic complicatioms with implant overdentures: A systematic literature review. Int. J. Prosthodont..

[36] Spazzin A.O., Costa A.R., Correr A.B., Consani R.L., Correr-Sobrinho L., dos Santos M.B. (2013). Effect of bar cross-section geometry on stress distribution in overdenture-retaining system simulating horizontal misfit and bone loss. J. Biomech..

[37] Kunwarjeet S., Nidhi G., Vikram K., Ridhimaa G. (2013). Hader bar and clip attachment retained mandibular complete denture. BMJ Case Rep..

[38] Hussein M.O. (2013). Stress-strain distribution at bone-implant interface of two splinted overdenture systems using 3D finite element analysis. J. Adv. Prosthodont..

[39] Elkerdawy M.W., Radi I.A. (2011). Effect of dislodging forces on mandibular implant attachment-retained overdenture. Implant. Dent..

[40] Bertolini M.M., Del Bel Cury A.A., Pizzoloto L., Acapa I.R.H., Shibli J.A., Bordin D. (2019). Does traumatic occlusal forces lead to peri-implant bone loss? A systematic review. Braz. Oral Res..

[41] Sakka S., Baroudi K., Nassani M.Z. (2012). Factors associated with early and late failure of dental implants. J. Investig. Clin. Dent..

[42] Sadowsky S.J., Caputo A.A. (2004). Stress transfer of four mandibular implant overdenture cantilever design. J. Prosthet. Dent..

[43] El-Anwar M., Ghali R., Aboelnagga M. (2016). 3D finite element study on: Bar splinted implants supporting partial denture in the reconstructed mandible. Maced. J. Med. Sci..

[44] Oh W.S., Saglik B., Bak S.Y. (2020). Bone loss in the posterior edentulous mandible with implant-supported overdentures vs complete dentures: A systematic review and meta-analysis. Int. J. Prosthodont..

[45] Stellinqsma K.J., Bouma J., Stegenga B., Meijer H.J., Raghoebar G.M. (2003). Satisfaction and psychosocial aspects of patients with an extremely resorbed mandible treated with implant retained overdentures. A prospective comparative study. Clin. Oral Implants Res..

[46] Pammer D. (2019). Evaluation of postoperative dental implant primary stability using 3D finite element analysis. Comput. Methods Biomech. Biomed. Eng..

[47] Chang Y., Tambe A.A., Maeda Y., Wada M., Gonda T. (2018). Finite element analysis of dental implants with validation: To what extent can we expect the model to predict biological phenomena? A literature review and proposal for classification of a validation process. Int. J. Implant Dent..

